# Tobacco Exposure During Pregnancy and Infections in Infants up to 1 Year of Age: The Japan Environment and Children’s Study

**DOI:** 10.2188/jea.JE20210405

**Published:** 2023-10-05

**Authors:** Koichi Hashimoto, Hajime Maeda, Hajime Iwasa, Hyo Kyozuka, Ryo Maeda, Yohei Kume, Takashi Ono, Mina Chishiki, Akiko Sato, Yuka Ogata, Tsuyoshi Murata, Keiya Fujimori, Kosei Shinoki, Hidekazu Nishigori, Seiji Yasumura, Mitsuaki Hosoya, the Japan Environment and Children’s Study (JECS) Group

**Affiliations:** 1 Fukushima Regional Center for the Japan Environmental and Children’s Study, Fukushima, Japan; 2 Department of Pediatrics, School of Medicine, Fukushima Medical University, Fukushima, Japan; 3 Department of Public Health, School of Medicine, Fukushima Medical University, Fukushima, Japan; 4 Department of Obstetrics and Gynecology, School of Medicine, Fukushima Medical University, Fukushima, Japan; 5 Fukushima Medical Center for Children and Women, Fukushima Medical University, Fukushima, Japan

**Keywords:** tobacco exposure, cohort study, pregnancy, child, infection

## Abstract

**Background:**

Tobacco exposure during pregnancy is associated with several adverse outcomes in infants. We investigated the association between tobacco exposure during pregnancy (both active and second-hand) and various infections in infants up to 1 year.

**Methods:**

This prospective cohort study used a fixed dataset (jecs-an-20180131) from the Japan Environment and Children’s Study of registered births in Japan during 2011–2014 that included 104,065 fetal records from enrolled pregnant women. Based on the participants’ responses to the questionnaire on smoking status, mothers were first divided into “never smoked,” “quit smoking,” and “current smoker” groups and then into “no second-hand smoking (SHS)” and “SHS” groups. Infectious diseases included central nervous system infection, otitis media (OM), upper respiratory tract infection (URTI), lower respiratory tract infection (LRTI), gastroenteritis (GI), and urinary tract infection. Adjusted odds ratios (aORs) and 95% confidence intervals (CIs) were calculated using logistic regression analysis and adjusted for maternal, socioeconomic, and postnatal confounding factors.

**Results:**

Among the 73,205 newborns enrolled, multivariable analysis revealed that the aOR of LRTI and GI was 1.20 (95% CI, 1.07–1.33) and 1.18 (95% CI, 1.04–1.35), respectively, for the “current smoker with/without SHS” group compared with the “never smoked without SHS” group. “Quit smoking without SHS” was not associated with the risk of LRTI. SHS was associated with an increased risk of OM, URTI, LRTI, and GI, especially with LRTI and GI.

**Conclusion:**

Exposure to tobacco smoke during pregnancy was associated with an increased risk of OM, URTI, LRTI, and GI in infants during their first year of life.

## INTRODUCTION

Morbidity and mortality from infectious diseases (IDs) have decreased worldwide; however, IDs remain a serious public health concern, especially for infants.^1^ In the United States, approximately 43% of all infant hospitalizations in 2003 were due to IDs.^2^ In Japan, respiratory tract infections are the most common reason children under 1 year of age visit hospitals or clinics, except for vaccinations and routine medical examinations. The estimated number of people visiting a medical institution for respiratory tract infections is similar to those visiting for vaccinations or infant medical examinations in Japan.^3^ Active maternal smoking is associated with multiple adverse outcomes, including low birth weight and preterm birth. Furthermore, maternal smoking, before and after birth, increases the risk of respiratory IDs in infants^4^^,^^5^ and is associated with other specific infections, not limited to the lungs.^6^ Smoking during pregnancy is associated with infant middle ear disease,^4^^,^^7^^,^^8^ bacterial meningitis,^9^^,^^10^ and gastroenteritis (GI).^11^^,^^12^ However, only a few studies have investigated the effects of maternal smoking on a broad range of respiratory and non-respiratory IDs in infants. Most of these reports targeted severe cases requiring hospitalization. A meta-analysis of 43 countries and statistical modeling of 131 countries revealed a global prevalence of smoking during pregnancy of 1.7% (95% confidence interval [CI], 0.0–4.5),^13^ but this varies between countries and regions. In Australia, 1 in 10 women (9.9%) who gave birth in 2017 smoked at some point during pregnancy.^14^ In Japan, the smoking prevalence among adult females peaked around the year 2000 and has been declining recently: 10% in 2000, 5% in 2010,^15^ and 2.7% in 2017.^16^ However, among young pregnant women, this rate is higher than in other age groups.^17^ Second-hand smoking (SHS) is another important source of tobacco exposure for pregnant women and children. They are exposed to smoke-containing environments in public places where smoking is allowed (workplaces, smokers’ homes, and vehicles).^18^ As of 2004, 40% of children and 35% of female non-smokers were exposed to SHS, according to data from the World Health Organization from 192 countries.^19^

Herein, we examined the association between tobacco exposure during pregnancy and various ID incidences up to 1 year after birth, regardless of hospitalization and disease severity, using data from a Japanese birth cohort study—the Japan Environment and Children’s Study (JECS).^20^^,^^21^ An actively smoking mother and a mother exposed to SHS during pregnancy both constitute tobacco exposure risk for infants. We investigated the association between active smoking and SHS during pregnancy and IDs in infants up to 1 year.

## METHODS

### Study design and population

The study protocol for JECS, a nationwide, government-funded prospective birth cohort study, has been reported.^20^ Participating pregnant women were registered between January 2011 and March 2014 in 15 regions throughout Japan. A total of 104,065 fetal records from pregnant women, including those with multiple pregnancies, were registered. The JECS protocol was reviewed and approved by the Ministry of the Environment’s Institutional Review Board on Epidemiological Studies and the Ethics Committees of all participating institutions. Written informed consent was obtained from all participants. The JECS was conducted in accordance with the Declaration of Helsinki and institutional/national regulations and guidelines.

### Data collection

**Figure 1.**
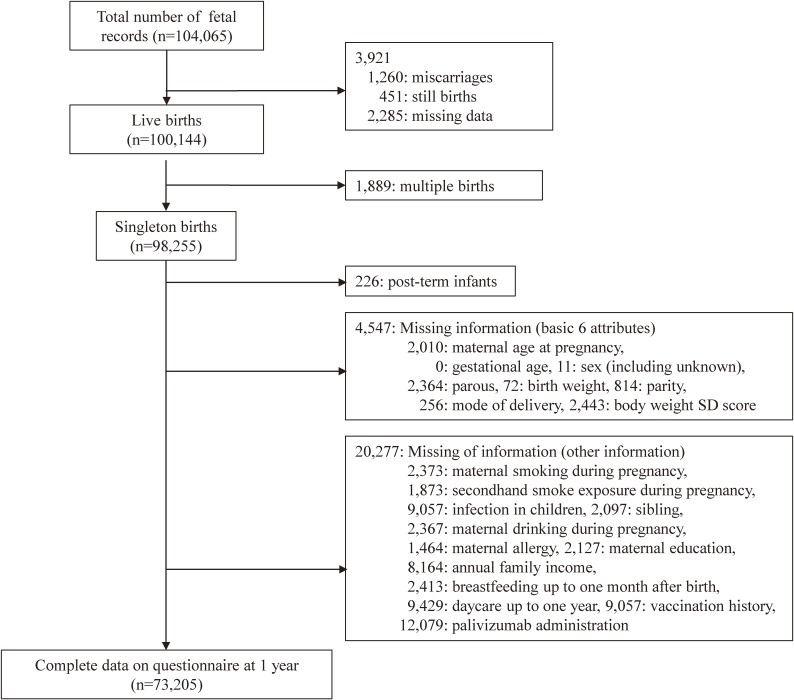
Study enrolment flowchart. A total of 104,065 fetal records of pregnant women, including those with multiple pregnancies, were included. After applying our criteria, of the 98,255 singleton births, 73,205 children without missing data for covariates were subjected to analysis. Because the “New Japanese neonatal anthropometric charts for gestational age at birth” used to define small for gestational age (SGA) cover gestational age (GA) 22–41 weeks, post-term infants (those over 41 weeks) were excluded.

This study used the jecs-an-20180131 dataset, released in March 2018. Data were collected from pregnancy to 1 year after delivery. We used five types of data from this dataset: (1) Dr-T1: examination records during the first trimester from co-operating health care providers including obstetricians and gynecologists; (2) M-T2: self-report questionnaires obtained from the mothers during the second or third trimester; (3) Dr-0m: examination records obtained from co-operating health care providers, including obstetricians and gynecologists, at the time of delivery; (4) M-1m: self-report questionnaires obtained from the mothers 1 month after delivery; and (5) C-1y: self-report questionnaires obtained from the caregiver 12 months after delivery. These five types of data were associated with the maternal ID and analyzed. We excluded cases with miscarriages, stillbirths, multiple pregnancies (Dr-0m), post-term infants, and missing responses related to covariance. After applying our criteria, of the 98,255 singleton births in the cohort, 73,205 non-post-term children without missing data for covariates were subjected to analysis (Figure 1).

### Outcomes, exposures, and covariates

ID information was obtained from the C-1y response to, “Has your child ever been diagnosed by a doctor with the following infections?” The following diseases were targeted regardless of hospitalization: encephalitis/encephalopathy, bacterial meningitis, viral meningitis/aseptic meningitis, otitis media (OM), upper respiratory tract infection (URTI; cold, pharyngitis), lower respiratory tract infection (LRTI; bronchitis, pneumonia), GI (viral GI), urinary tract infections (UTI), pertussis, herpangina, hand-foot-and-mouth disease, adenovirus infection, and respiratory syncytial virus (RSV) infection. Furthermore, some diseases were grouped as follows: encephalitis/encephalopathy, bacterial meningitis, and viral meningitis/aseptic meningitis were classified as central nervous system infections (CNSI); pertussis, herpangina, hand-foot-and-mouth disease, and adenovirus infection were classified as URTI; and RSV infection was classified as LRTI.

Maternal smoking was defined as the mother’s active smoking, and SHS was defined as exposure to the smoke of cigarettes smoked by others. Maternal smoking status (never smoked, quit before pregnancy, quit after pregnancy, current smoker) was obtained at M-T2; the questionnaire was designed as follows: 1 = never; 2 = previously did, but quit before recognizing current pregnancy; 3 = previously did, but quit after finding out about current pregnancy; 4 = yes, I still smoke. “Quit before pregnancy” and “quit after pregnancy” were combined as “quit smoking,” and smoking status was reclassified into three categories: “never smoked,” “quit smoking,” and “current smoker.” Furthermore, “current smoker” was divided into three groups based on the average cigarettes smoked daily: 0–5, 6–10, and 11–40. SHS status (“No SHS” and “SHS”) was obtained at M-T2: second-hand smoke exposure (yes, no). Furthermore, pregnant women were categorized into five groups based on their tobacco exposure: “never smoked without exposure to SHS,” “never smoked with exposure to SHS,” “quit smoking without exposure to SHS,” “quit smoking with exposure to SHS,” and “current smoker with/without exposure to SHS.”

The prevalence of IDs in infancy is related to antenatal and perinatal maternal, socioeconomic maternal, and postnatal backgrounds.^5^ Herein, in addition to maternal background factors, postnatal background factors were adjusted for. The definition and classification of confounding factors as well as details of the management of selection bias are available in eMaterial 1 (Supplementary methods).

### Statistical analyses

Participant characteristics based on prenatal smoking status were calculated as percentages. All statistical analyses were performed using Stata/SE14 (StataCorp LP, College Station, TX, USA). We calculated unadjusted and adjusted odds ratios (aORs) and 95% CIs for IDs based on maternal tobacco exposure status and number of cigarettes smoked using logistic regression analysis. Crude odds ratios (cORs) were estimated for the association between maternal tobacco exposure status and IDs in infants up to 1 year of age using the reference category of “never smoked without exposure to SHS” (model 1). Next, we estimated aORs for models 2 and 3 by sequentially adjusting for perinatal and socioeconomic factors and postnatal factors. In addition, we estimated cORs for the association between the number of cigarettes smoked in “current smoker” and IDs, using the reference category “never smoked” (model 1). Thereafter, aORs were estimated for models 2–4 by sequentially adjusting for SHS, perinatal and socioeconomic factors, and postnatal factors. Detailed methods for evaluating selection bias are given in eMaterial 1 (Supplementary methods).

## RESULTS

### Antenatal and prenatal, socioeconomic, and postnatal background and infection outcomes in children

Comparison of the basic attributes between the analyzed (*n* = 73,205) and data-deficient groups (*n* = 20,277) was performed using the chi-square test (see eMaterial 1 (Supplementary methods)). The phi coefficients in all comparisons of basic attributes were less than 0.1 (Table 1).

**Table 1.** Comparison of basic attributes of the analysis and data-deficient groups

	Analysis group		Data-deficient group		*P*-value	phi
	
	73,205		20,277			
Maternal age at pregnancy, years						
≤29	28,427	38.8%	9,758	48.1%	<0.001	0.0779
≥30	44,778	61.2%	10,519	51.9%		
Parity						
Primiparous	21,718	29.7%	5,466	27.0%	<0.001	0.0246
Multiparous	51,487	70.3%	14,811	73.0%		
Sex						
Male	37,467	51.2%	10,434	51.5%	0.486	0.0023
Female	35,738	48.8%	9,843	48.5%		
Gestational age, weeks						
≤36	3,204	4.4%	1,187	5.9%	<0.001	0.0288
≥37	70,001	95.6%	19,090	94.1%		
Cesarean section						
Yes	13,512	18.5%	4,020	19.8%	<0.001	0.0144
No	59,693	81.5%	16,257	80.2%		
SGA						
Yes	2,520	3.4%	813	4.0%	<0.001	0.0126
No	70,685	96.6%	19,464	96.0%		

SGA, small for gestational age.

For the evaluation of selection bias in the analysis group, the data-deficient group in which any one or more of the exposures, outcomes, and covariates were missing was compared with the analysis group using the *χ*-square test for basic attributes; both groups had data with all basic attributes. The basic attributes were binarized as follows: maternal age (≤29, ≥30 years), parity (primiparous, multiparous), infant sex (male, female), gestational age (≤36, ≥37 weeks), cesarean section (yes, no), and SGA (yes, no). A *P*-value <0.05 was regarded as statistically significant. The difference between the two groups was evaluated using the phi coefficient. Interpretations regarding the effect size of phi coefficients corresponded to a previously stated criterion, that is, correlations of approximately 0.1 are weak, 0.3 are moderate, and 0.5 are strong.^47^

Table 2 shows participant characteristics according to maternal smoking status. Regarding maternal smoking status, women in “never smoked,” “quit smoking,” and “current smoker” groups comprised 59.4%, 36.8%, and 3.8% of the participants, respectively. The proportions with various antenatal and perinatal backgrounds, including socioeconomic and postnatal backgrounds, are presented. The proportion of children with one or more IDs was 47.6% for “overall,” 47.0% for “never smoked,” 48.3% for “quit smoking,” and 50.2% for “current smoker.”

**Table 2.** Participant characteristics according to maternal smoking status during pregnancy

	Total		Never smoked		Quit smoking		Current smoker	
			
	73,205	100%	43,452	59.4%	26,948	36.8%	2,805	3.8%
	*n*	%	*n*	%	*n*	%	*n*	%
**Antenatal and perinatal backgrounds**								
Maternal age at pregnancy, years								
<20	463	0.6%	242	0.6%	181	0.7%	40	1.4%
20–29	27,964	38.2%	16,085	37.0%	10,575	39.2%	1,304	46.5%
30–39	42,179	57.6%	25,506	58.7%	15,302	56.8%	1,371	48.9%
≥40	2,599	3.6%	1,619	3.7%	890	3.3%	90	3.2%
Parity								
0	21,718	29.7%	14,607	33.6%	6,647	24.7%	464	16.5%
1	25,389	34.7%	15,470	35.6%	9,193	34.1%	726	25.9%
≥2	26,098	35.7%	13,375	30.8%	11,108	41.2%	1,615	57.6%
Sex, male	37,467	51.2%	22,313	51.4%	13,698	50.8%	1,456	51.9%
Gestational age, weeks								
≤28	104	0.1%	60	0.1%	40	0.1%	4	0.1%
29–34	761	1.0%	414	1.0%	307	1.1%	40	1.4%
35–36	2,339	3.2%	1,356	3.1%	862	3.2%	121	4.3%
37–41	70,001	95.6%	41,622	95.8%	25,739	95.5%	2,640	94.1%
SGA	2,520	3.4%	1,440	3.3%	879	3.3%	201	7.2%
Caesarian section	13,512	18.5%	7,744	17.8%	5,198	19.3%	570	20.3%
Maternal allergy	37,701	51.5%	22,344	51.4%	14,002	52.0%	1,355	48.3%
Second-hand smoking	26,276	35.9%	12,082	27.8%	11,818	43.9%	2,376	84.7%
Alcohol drinking	2,042	2.8%	798	1.8%	981	3.6%	263	9.4%
**Socioeconomic background**								
Maternal education, years								
<10	2,800	3.8%	525	1.2%	1,665	6.2%	610	21.7%
10–12	23,022	31.4%	10,736	24.7%	10,866	40.3%	1,420	50.6%
13–16	46,234	63.2%	31,227	71.9%	14,236	52.8%	771	27.5%
≥17	1,149	1.6%	964	2.2%	181	0.7%	4	0.1%
Annual family income, Japanese yen								
<2,000,000	3,747	5.1%	1,581	3.6%	1,758	6.5%	408	14.5%
2,000,000–5,999,999	49,522	67.6%	28,124	64.7%	19,372	71.9%	2,026	72.2%
6,000,000–9,999,999	16,774	22.9%	11,544	26.6%	4,921	18.3%	309	11.0%
≥10,000,000	3,162	4.3%	2,203	5.1%	897	3.3%	62	2.2%
**Postnatal backgrounds**								
Sibling	40,522	55.4%	23,195	53.4%	15,581	57.8%	1,746	62.2%
Breastfeeding at 1 month	72,270	98.7%	43,028	99.0%	26,559	98.6%	2,683	95.7%
Nursery at 1 year	20,003	27.3%	11,151	25.7%	7,838	29.1%	1,014	36.1%
Vaccines								
DPT vaccine	65,981	90.1%	39,450	90.8%	24,113	89.5%	2,418	86.2%
Hib vaccine	70,069	95.7%	41,773	96.1%	25,709	95.4%	2,587	92.2%
Pneumococcal vaccine	68,772	93.9%	41,073	94.5%	25,192	93.5%	2,507	89.4%
Rotavirus vaccine	31,918	43.6%	20,418	47.0%	10,845	40.2%	655	23.4%
Palivizumab	1,907	2.6%	1,064	2.4%	752	2.8%	91	3.2%
**Infection outcomes**								
Infection	34,839	47.6%	20,415	47.0%	13,015	48.3%	1,409	50.2%
CNSI	110	0.2%	57	0.1%	48	0.2%	5	0.2%
OM	8,638	11.8%	4,928	11.3%	3,303	12.3%	407	14.5%
URI	23,439	32.0%	13,989	32.2%	8,568	31.8%	882	31.4%
LRI	10,774	14.7%	6,064	14.0%	4,184	15.5%	526	18.8%
GI	6,899	9.4%	3,761	8.7%	2,798	10.4%	340	12.1%
UTI	548	0.7%	338	0.8%	194	0.7%	16	0.6%

CNSI, central nervous system infection; DPT, diphtheria, pertussis, and tetanus; GI, gastrointestinal infection; Hib, *Haemophilus influenzae* type b; LRTI, lower respiratory tract infection; OM, otitis media; SGA, small for gestational age; URTI, upper respiratory tract infection; UTI, urinary tract infection.

Pregnant women were categorized into three groups based on their smoking status: “never smoked,” “quit smoking,” and “current smoker.” Infections include CNSI, OM, URTI, LRTI, GI, and UTI.

eTable 1 shows participant characteristics according to maternal tobacco exposure status. Women in “never smoked without exposure to SHS” (Group 1), “never smoked with exposure to SHS” (Group 2), “quit smoking without exposure to SHS” (Group 3), “quit smoking with exposure to SHS” (Group 4), and “current smoker with/without exposure to SHS” (Group 5) groups comprised 42.9%, 16.5%, 20.7%, 16.1%, and 3.8% of the participants, respectively. The proportions of children with one or more IDs were 46.5%, 48.3%, 47.8%, 48.9%, and 50.2% for Group 1, Group 2, Group 3, Group 4, and Group 5, respectively.

### Infection risk during the first year of life

Table 3 shows the association between tobacco exposure during pregnancy and IDs in children up to 1 year. Before adjusting for covariates, children of the members of “current smoker with/without SHS” had an increased risk of infant OM, LRTI, and GI morbidity compared with those of “never smoked without SHS,” with cORs of 1.39 (95% CI, 1.24–1.55), 1.46 (95% CI, 1.32–1.62), and 1.54 (95% CI, 1.37–1.74), respectively. Children of mothers from the “quit smoking without SHS” group also had increased risks of OM, LRTI, and GI, with cORs of 1.09 (95% CI, 1.03–1.16), 1.10 (95% CI, 1.04–1.16), and 1.21 (95% CI, 1.13–1.30), respectively (model 1). After adjusting for prenatal, perinatal, and socioeconomic factors, active smoking increased the risks of OM, URTI, LRTI, and GI, with aORs of 1.26 (95% CI, 1.12–1.41), 1.14 (95% CI, 1.04–1.24), 1.30 (95% CI, 1.17–1.44), and 1.35 (95% CI, 1.19–1.53), respectively. Previous smoking without SHS increased the risk of GI, with an aOR of 1.15 (95% CI, 1.08–1.24) (model 2). Finally, active smoking was associated with an elevated risk of LRTI and GI, even after adjusting for all covariates, with aORs of 1.20 (95% CI, 1.07–1.33) and 1.18 (95% CI, 1.04–1.35), respectively. Previous smoking without SHS was not associated with LRTI risk, with an aOR of 1.01 (95% CI, 0.95–1.07), but increased the risk of GI, with an aOR of 1.12 (95% CI, 1.05–1.20). Current smoking had no association with increased risks of CNSI, OM, URTI, and UTI (model 3).

**Table 3.** Relationship between tobacco exposure during pregnancy and infection in infants up to 1 year of age

Infections	Smoking status		Model 1		Model 2		Model 3	
	Maternal smoking	Second-hand smoking	cOR	95% CI	aOR	95% CI	aOR	95% CI
Infections	Never	+	1.07	1.03–1.12	1.14	1.09–1.19	1.06	1.02–1.11
	Quit	−	1.05	1.01–1.10	1.04	1.00–1.09	1.02	0.98–1.06
	Quit	+	1.10	1.06–1.15	1.15	1.10–1.21	1.08	1.03–1.13
	Current	−/+	1.16	1.08–1.26	1.21	1.11–1.31	1.11	1.02–1.21

CNSI	Never	+	1.40	0.81–2.42	1.45	0.84–2.52	1.40	0.81–2.43
	Quit	−	1.57	0.96–2.57	1.55	0.94–2.55	1.53	0.93–2.53
	Quit	+	1.44	0.83–2.47	1.41	0.80–2.50	1.36	0.77–2.41
	Current	−/+	1.51	0.59–3.85	1.40	0.52–3.76	1.31	0.49–3.51

OM	Never	+	1.16	1.09–1.24	1.20	1.12–1.28	1.07	1.00–1.15
	Quit	−	1.09	1.03–1.16	1.04	0.97–1.10	0.99	0.93–1.06
	Quit	+	1.21	1.13–1.29	1.17	1.09–1.25	1.03	0.96–1.10
	Current	−/+	1.39	1.24–1.55	1.26	1.12–1.41	1.08	0.96–1.22

URTI	Never	+	1.03	0.99–1.08	1.12	1.07–1.17	1.06	1.01–1.11
	Quit	−	1.00	0.96–1.04	1.03	0.98–1.07	1.00	0.96–1.05
	Quit	+	0.98	0.94–1.03	1.10	1.05–1.15	1.04	0.99–1.09
	Current	−/+	0.97	0.90–1.06	1.14	1.04–1.24	1.07	0.98–1.17

LRTI	Never	+	1.10	1.04–1.17	1.12	1.05–1.19	1.04	0.98–1.11
	Quit	−	1.10	1.04–1.16	1.03	0.97–1.09	1.01	0.95–1.07
	Quit	+	1.26	1.19–1.33	1.19	1.12–1.27	1.11	1.04–1.18
	Current	−/+	1.46	1.32–1.62	1.30	1.17–1.44	1.20	1.07–1.33

GI	Never	+	1.22	1.14–1.31	1.21	1.12–1.30	1.11	1.03–1.19
	Quit	−	1.21	1.13–1.30	1.15	1.08–1.24	1.12	1.05–1.20
	Quit	+	1.41	1.31–1.51	1.31	1.22–1.41	1.19	1.11–1.29
	Current	−/+	1.54	1.37–1.74	1.35	1.19–1.53	1.18	1.04–1.35

UTI	Never	+	1.02	0.80–1.29	1.03	0.81–1.31	1.03	0.81–1.31
	Quit	−	0.89	0.70–1.12	0.88	0.69–1.11	0.87	0.69–1.10
	Quit	+	0.98	0.77–1.25	0.99	0.76–1.27	0.98	0.76–1.26
	Current	−/+	0.73	0.44–1.22	0.71	0.42–1.20	0.70	0.41–1.19

aOR, adjusted odds ratio; CI, confidence interval; CNSI, central nervous system infection; cOR, crude odds ratio; DPT, diphtheria, pertussis, and tetanus; GI, gastrointestinal infection; Hib, *Haemophilus influenzae* type b; LRTI, lower respiratory tract infection; OM, otitis media; SGA, small for gestational age; URTI, upper respiratory tract infection; UTI, urinary tract infection.

Pregnant women were categorized into five groups based on their tobacco exposure: “never smoked without exposure to SHS (Never, −),” “never smoked with exposure to SHS (Never, +),” “quit smoking without exposure to SHS (Quit, −),” “quit smoking with exposure to SHS (Quit, +),” and “current smoker (Current, −/+).”

The ORs generated by pregnant women’s smoking status were calculated using logistic regression analysis compared to “Never, −.” Infections include CNSI, OM, URTI, LRTI, GI, and UTI.

Model 1: unadjusted model.

Model 2: model 1 adjusted for antenatal, perinatal, and socioeconomic backgrounds: maternal age at pregnancy, parity, sex (male), gestational age (weeks), SGA, cesarean section, maternal allergy, alcohol drinking habits, maternal education (years), and annual family income (JPY).

Model 3: model 3 adjusted for postnatal background, sibling, breastfeeding at 1 month, and daycare experience up to 1 year of age.

SHS increased the risk of infant OM, LRTI, and GI morbidity compared with “without SHS” in each active maternal smoking status. The addition of SHS in “never smoked” and “quit smoking” increased the risk of these infections (model 1). In OM and URTI, the risk was not significantly increased in “quit smoking” and “current smoker,” but the risk of OM, URTI, and GI increased with the addition of SHS in “never smoked,” with aORs of 1.07 (95% CI, 1.00–1.15, *P* = 0.043), 1.06 (95% CI, 1.01–1.11), and 1.11 (95% CI, 1.03–1.19), respectively. Furthermore, SHS increased the risk of LRTI and GI in “quit smoking” (model 3).

Table 4 shows the association between the average number of cigarettes smoked daily and IDs in children up to 1 year of age. Approximately 4% of pregnant women continued to smoke (Table 2), and the maximum average number of cigarettes smoked daily was 40. “Current smoker” was categorized into three groups—those who smoked 0–5 cigarettes/day (37.8% of current smokers), 6–10/day (42.4%), and 11–40/day (19.9%). The cORs of the heavy smokers (11–40/day) were higher than those of other groups for OM, LRTI, and GI, being 1.51 (95% CI, 1.20–1.90), 1.56 (95% CI, 1.26–1.92), and 1.99 (95% CI, 1.58–2.50), respectively. After adjusting for SHS, prenatal, perinatal, socioeconomic, and postnatal factors (model 4), children of the lightest smokers (0–5/day) and the heavy smokers had increased risks of LRTI and GI, with aORs of 1.21 (95% CI, 1.03–1.44) and 1.44 (95% CI, 1.12–1.85), respectively.

**Table 4.** Relationship between the average number of cigarettes smoked daily in current smokers and infection in infants up to 1 year of age

Infection	No. of cigarettes per day	Model 1	Model 2	Model 3	Model 4
		cOR (95% CI)	aOR (95% CI)	aOR (95% CI)	aOR (95% CI)
Infections	Never smoked	ref.	ref.	ref.	ref.
	0–5	1.14 (1.01–1.29)	1.10 (0.97–1.24)	1.10 (0.97–1.25)	1.09 (0.96–1.25)
	6–10	1.08 (0.96–1.21)	1.04 (0.92–1.17)	1.00 (0.88–1.13)	0.98 (0.86–1.11)
	11–40	1.27 (1.07–1.50)	1.22 (1.03–1.44)	1.20 (1.01–1.43)	1.16 (0.97–1.40)
CNSI	Never smoked	ref.	ref.	ref.	ref.
	0–5	1.45 (0.35–5.93)	1.19 (0.28–5.01)	1.07 (0.24–4.76)	1.04 (0.24–4.59)
	6–10	0.64 (0.09–4.66)	0.52 (0.07–3.84)	0.47 (0.06–3.60)	0.44 (0.06–3.39)
	11–40	2.75 (0.67–11.31)	2.19 (0.52–9.29)	1.84 (0.38–8.83)	1.77 (0.37–8.49)
OM	Never smoked	ref.	ref.	ref.	ref.
	0–5	1.28 (1.07–1.52)	1.18 (0.98–1.41)	1.07 (0.89–1.29)	1.03 (0.85–1.24)
	6–10	1.28 (1.08–1.51)	1.17 (0.99–1.39)	1.02 (0.86–1.22)	0.97 (0.81–1.17)
	11–40	1.51 (1.20–1.90)	1.38 (1.09–1.73)	1.20 (0.95–1.53)	1.12 (0.87–1.44)
URTI	Never smoked	ref.	ref.	ref.	ref.
	0–5	0.98 (0.86–1.12)	0.96 (0.84–1.10)	1.06 (0.92–1.22)	1.05 (0.91–1.21)
	6–10	0.91 (0.80–1.03)	0.89 (0.79–1.01)	0.96 (0.84–1.10)	0.95 (0.83–1.09)
	11–40	1.06 (0.89–1.27)	1.04 (0.87–1.24)	1.17 (0.97–1.41)	1.14 (0.94–1.39)
LRTI	Never smoked	ref.	ref.	ref.	ref.
	0–5	1.42 (1.22–1.67)	1.35 (1.15–1.58)	1.22 (1.03–1.44)	1.21 (1.03–1.44)
	6–10	1.35 (1.16–1.57)	1.27 (1.09–1.48)	1.10 (0.94–1.29)	1.08 (0.92–1.27)
	11–40	1.56 (1.26–1.92)	1.46 (1.18–1.81)	1.28 (1.02–1.59)	1.23 (0.98–1.54)
GI	Never smoked	ref.	ref.	ref.	ref.
	0–5	1.47 (1.22–1.77)	1.33 (1.10–1.60)	1.21 (0.99–1.47)	1.16 (0.95–1.42)
	6–10	1.19 (0.98–1.44)	1.06 (0.87–1.29)	0.95 (0.78–1.16)	0.90 (0.73–1.10)
	11–40	1.99 (1.58–2.50)	1.76 (1.39–2.22)	1.54 (1.20–1.97)	1.44 (1.12–1.85)
UTI	Never smoked	ref.	ref.	ref.	ref.
	0–5	0.61 (0.25–1.47)	0.60 (0.24–1.46)	0.63 (0.26–1.56)	0.61 (0.25–1.52)
	6–10	0.76 (0.36–1.61)	0.74 (0.35–1.60)	0.78 (0.36–1.70)	0.76 (0.35–1.67)
	11–40	0.93 (0.34–2.49)	0.91 (0.33–2.46)	0.99 (0.35–2.78)	0.96 (0.34–2.70)

aOR, adjusted odds ratio; CI, confidence interval; CNSI, central nervous system infection; cOR, crude odds ratio; DPT, diphtheria, pertussis, and tetanus; GI, gastrointestinal infection; Hib, *Haemophilus influenzae* type b; LRTI, lower respiratory tract infection; OM, otitis media; ref, reference; URTI, upper respiratory tract infection; UTI, urinary tract infection.

Of the 2,805 current smokers, 2,793 were analyzed, excluding 12 who smoked unknown numbers of cigarettes. Current smokers, for whom the data for the number of cigarettes smoked per day was not missing, were categorized into three groups based on the average daily number of cigarettes smoked: 0–5 (*n* = 1,055), 6–10 (*n* = 1,183), 11–40 (*n* = 555) cigarettes per day. OR was calculated using logistic regression analysis compared with never smoked. Infections include CNSI, OM, URTI, LRTI, GI, and UTI.

Model 1: unadjusted model.

Model 2: model 1 adjusted for SHS.

Model 3: model 2 adjusted for antenatal, perinatal, and socioeconomic backgrounds: maternal age at pregnancy, parity, sex (male), gestational age (weeks), SGA, cesarean section, maternal allergy, alcohol drinking habits, maternal education (years), and annual family income (JPY).

Model 4: model 3 adjusted for postnatal background, sibling, breastfeeding at 1 month, daycare experience up to 1 year of age, DPT vaccine, Hib vaccine, pneumococcal vaccine, rotavirus vaccine, and palivizumab administration.

## DISCUSSION

Maternal tobacco exposure during pregnancy increased the risk of LRTI and GI during the first year in a model adjusted for maternal, socioeconomic, and postnatal factors. However, when pregnant women quit smoking before or after noticing their pregnancy without SHS the risk of LRTI in infants during the first year did not differ from that in infants born to non-smokers without SHS. Maternal SHS was associated with increased risk of OM, URTI, LRTI, and GI. The odds ratios for the risk of infections in infants due to maternal smoking and SHS adjusted for various factors were not high in this study with a large sample size. However, increased risks even when adjusted for various factors imply that tobacco exposure during pregnancy is intimately involved in the risk of infections in infants.

A systemic review and meta-analysis on parental smoking and the risk of OM has reported the following.^22^ Prenatal maternal smoking (OR 1.11; 95% CI, 0.93–1.31) was not associated with the risk of OM, as opposed to postnatal smoking by the mother (OR 1.62; 95% CI, 1.33–1.97) or any household member (OR 1.37; 95% CI, 1.25–1.50). Our analysis supports this report, as the maternal smoking status during pregnancy and number of cigarettes were not associated with increased risk of developing OM in children. Active maternal smoking was not associated with the risk of URTI up to 6 months of age^23^ or in children aged 8–12 years,^24^ with aORs of 0.80 (95% CI, 0.58–1.09) and 0.77 (95% CI, 0.57–1.03), respectively. However, active maternal smoking during pregnancy and after birth^23^ or exposure of children to tobacco from birth to 2 years of age^24^ is associated with increased risk of URTI in children up to 6 months of age or between 8 and 12 years of age, with aORs of 1.58 (95% CI, 1.07–2.35) and 1.25 (95% CI, 1.10–1.41), respectively. We also showed that active smoking by pregnant mothers was not associated with URTI risk in a fully adjusted model (model 3). However, the passive smoking environment was associated with an increased risk of OM and URTI in non-smokers, albeit slightly, with aORs of 1.07 (95% CI, 1.00–1.15, *P* = 0.043) and 1.06 (95% CI, 1.01–1.11), respectively. The reasons for the increased OM and URTI risk are unknown, although there is no effect of SHS on LRTIs, which is more closely associated with tobacco exposure during pregnancy, in non-smokers. This may be related to the amount of SHS exposure during pregnancy or after birth.

After adjustment for maternal factors during pregnancy, the increased risk of LRTI in infants was 1.46 in the “current smoker” group, and adjusting for socioeconomic and postnatal factors generated ORs of 1.30 and 1.20, respectively. In a systematic review, prenatal maternal smoking was associated with LRTI risk, with an OR of 1.24 (95% CI, 1.11–1.38).^25^ Therefore, the LRTI risk at full adjustment in this study was similar to that previously reported, supporting an increased risk of LRTI in infants due to maternal smoking during pregnancy. Furthermore, in this analysis, the risk of infant LRTI was increased by active maternal smoking, but only for the lightest smokers (0–5 cigarettes/day), suggesting the uncertainty and limitations of self-administered surveys. Moreover, previous smoking without SHS was not associated with LRTI risk, underlining the importance of smoking cessation among pregnant women and non-tobacco exposure environment.

Maternal smoking is a risk factor for GI in children below 12 months of age; it increases the risk of infant hospitalization due to viral GI (OR 1.2; 95% CI, 1.1–1.4).^11^ Furthermore, environmental tobacco smoke exposure was significantly associated with acute GI (relative risk 2.25; 95% CI, 1.26–5.18).^26^ The major viruses causing GI in infants are rotavirus, adenovirus, norovirus, and sapovirus. Rotavirus infections can be prevented or mitigated by vaccines; however, vaccines for the other viruses are still under development. The main symptoms of GI are diarrhea and/or vomiting. However, it may be difficult to distinguish GI from any accompanying symptoms of common cold, formula intolerance, or food allergies. Without laboratory testing, the diagnosis of infectious GI, including mild cases, is often inaccurate. Herein, the greater the chance of tobacco exposure during pregnancy, the higher the infection risk in infants. However, the same risk of infectious disease in “quit smoking without SHS” and “never smoked with SHS” groups is presumably due to the effect of third-hand smoking on the increased GI risk. These facts suggest that tobacco exposure environment during pregnancy is associated with an increased risk of GI in infants.

The link between CNSI in infants and maternal smoking has been shown in meningitis.^9^^,^^27^ Meningitis due to meningococci, *Haemophilus influenzae*, and enteroviruses is associated with parental smoking, having older siblings, and low birth weight in infants.^28^ Because meningitis due to meningococci is rare in Japan,^29^^,^^30^ and the causative agent of meningitis was unknown in this study, we could not analyze in detail the association between maternal smoking and meningitis and did not find any association between them.

UTIs in infants are often associated with congenital anomalies of the kidney and urinary tract (CAKUT), especially urinary tract anomalies. CAKUT comprises a broad spectrum of distinct phenotypes; however, little is known about risk factors in specific CAKUT phenotypes. In a Swedish study, smoking in early pregnancy was associated with kidney, but not urinary tract, anomalies.^31^ However, smoking was not found to increase the CAKUT risk in children.^32^ Furthermore, there was no association between smoking during pregnancy and UTI in the present study.

With regard to the relationship between the daily number of cigarettes smoked and ID risk in infants, the heaviest maternal smokers generated the highest cORs for OM, LRTI, and GI, suggesting a dose-response relationship in the pre-adjustment model (model 1). This tendency was also seen for GI in models 3 and 4.

In utero exposure to smoking affects respiratory function or immunity in infants and is associated with lower lung function levels in the neonatal period.^33^ Gilliland et al demonstrated significantly reduced lung function in children exposed to maternal smoking in utero.^34^ Studies analyzing cord blood in infants^35^^–^^37^ and exposure in primates^38^ have shown that exposure to cigarette smoke can alter the infants’ immune system. Regarding the possible mechanism underlying GI development, environmental tobacco smoke exposure might suppress the production of gut IgA, one of the key antimicrobial substances in the gut, because it suppresses the capacity of B cells to produce immunoglobulins.^39^ Another possibility is a direct toxic effect of tobacco products on the epithelial cells of the respiratory tract and gut, causing impaired first-line protection against invading microorganisms.^40^

SHS, including during pregnancy, increases the risk of adverse pregnancy and health outcomes during childhood, as well as infections. Smoking cessation laws significantly reduce the number of perinatal deaths, preterm births, and hospitalizations for respiratory tract infections and asthma in children.^41^^–^^46^ Improving children’s health through such legislation is likely to reduce smoking during pregnancy and exposure to SHS at home, in addition to protecting children from encountering smoke in public spaces. Here, 40.6% of all mothers, including current smokers, had smoked, and 35.9% were exposed to SHS. Furthermore, the SHS rates for non-smokers, previous smokers, and active smokers were 27.8%, 43.9%, and 84.7%, respectively, suggesting a relationship between maternal smoking and SHS. Therefore, even the slightly higher risk of OM, URTI, LRTI, and GI due to SHS in this analysis emphasizes the health benefits of avoiding smoky environments and providing smoking cessation education from childhood.

The strength of this study is that overall childhood infections, regardless of hospitalization and severity, were evaluated in a prospective, nationwide, cohort study. Multivariable analysis was performed, adjusting for maternal socioeconomic factors and postnatal factors, such as cohabitation of children under 1 year of age and vaccination status, in addition to maternal factors. Rotavirus causes GI, and *Haemophilus influenzae* type b (Hib), pneumococcus, and the pertussis pathogen cause CNSI, respiratory tract infection, and OM in children. The diseases caused by these pathogens are vaccine-preventable, and infants in Japan are vaccinated against them approximately 2 months after birth.^47^ In Japan, vaccinations against Hib and pneumococcus, and rotavirus became routine in 2013 and 2020, respectively. RSV is one of the most important viral pathogens causing acute LRTI in young children. In Japan, palivizumab administration to prevent severe RSV infection was started in 2002; however, the target child groups were changed in 2005 and 2013.^48^ Because the children analyzed in this study were born between 2011 and 2014, vaccination status and palivizumab administration were added as adjustment factors in the analysis.

This study has three limitations. First, we did not assess the smoking environment of children after birth and the extent of SHS. Second, there was no information available on hospitalization or severity of IDs. Most previous reports have targeted inpatients and severe cases, but we analyzed all patients regardless of inpatient or outpatient status for various IDs. Third, approximately 25% of singleton infants were in the data-deficient group, which may have caused selection bias in the analysis group. Although a very small difference was observed between the two groups, the effect size of phi coefficients was less than 0.1,^49^ and such a small difference is significant due to the large sample size and high detection power. Moreover, the answers provided on the questionnaires, especially for the questions on diagnosis, may not have been accurate, as the survey was completed by participants. Because maternal smoking status was obtained using self-report and a mother may hesitate to provide an accurate answer on her smoking status due to feeling guilty because of the child, smoking is likely to have been underreported; thus, our findings could underestimate the true prevalence of infant exposure to cigarette smoke.

In conclusion, tobacco exposure during pregnancy was associated with an increased risk of OM, URTI, LRTI, and GI in infants during their first year of life.

## SUPPLEMENTARY MATERIAL

eMaterial 1. Supplementary methods

eTable 1. Participant characteristics according to tobacco exposure during pregnancy
