# Supplementary material for: Tobacco Exposure During Pregnancy and Infections in Infants up to 1 Year of Age: The Japan Environment and Children’s Study
Source: J Epidemiol. 2023 Oct 5;33(10):489–97. doi: 10.2188/jea.JE20210405 (PMC10483106; doi:10.2188/jea.JE20210405)
Supplement: Supplementary file 1 [file je-33-489-s001.pdf]

eMaterial 1. Supplementary methods

Definition and classification of confounding factors

The data relating to these variables were obtained from the questionnaires Dr-T1, M-T2, Dr-0m, M-1m, and C-1y. Data for maternal age at delivery (<20, 20–29, 30–39, and ≥40 years), parity (primiparous, multiparous), and maternal allergies (yes, no) were obtained from Dr-T1. Data for maternal smoking status (never smoked, quit smoking, current smoker), second-hand smoke exposure (yes, no), maternal alcohol drinking habits (yes, no), maternal educational background (junior high school: <10; high school: 10–12; technical junior college or technical/vocational college: 13–16; graduate school: ≥17 years), and annual household income (<2 million, 2–6 million, 6–10 million, and ≥10 million Japanese yen) were obtained from M-T2. Data for infant's sex (male, female), gestational age (GA; ≤28, 29–34, 35–36, and 37–41 weeks), SGA (yes, no), and cesarean section (yes, no) were obtained from Dr-0m. SGA was defined as having a height and weight below −1.5 standard deviations at birth and was corrected for gestational age, sex, and primipara/multipara based on the "New Japanese neonatal anthropometric charts for gestational age at birth,"[45] which covered GA 22–41 weeks. Data for sibling (yes, no) and breastfeeding (yes, no) were obtained from M1m. Data for vaccination history (yes, no) for diphtheria, pertussis, and tetanus (DPT), *Haemophilus influenza* type b (Hib), pneumococcal, rotavirus vaccine, and palivizumab up to 1 year of age were obtained from C-1y. Additionally, data for daycare experience (yes, no) up to 1 year were obtained from C-1y.

We considered the following confounding factors to influence the data: maternal age, parity, infant sex, GA, SGA, cesarean section, maternal allergies, maternal smoking status, second-hand smoke exposure, maternal alcohol drinking habits, maternal educational background, annual household income, sibling, breastfeeding at 1 month of age, palivizumab administration,

vaccination history, and daycare experience.

Selection bias

To evaluate any selection bias in the analysis group, the basic attributes were binarized as follows: maternal age (≤29, ≥30 years), parity (primiparous, multiparous), infant sex (male, female), GA (≤36, ≥37 weeks), cesarean section (yes, no), and SGA (yes, no).

Each basic attribute was compared between the analysis group and the data-deficient group (defined as the group in which any one or more of the exposures, outcomes, and covariates were missing), using the χ-square test. The difference between the two groups was evaluated using the phi coefficient.

Comparison of basic attributes between the analysis group (n=73,205) and the data-deficient group (n=20,277) was performed using the χ-square test. The phi coefficients in all comparisons of basic attributes were less than 0.1 (Table 1).

eTable 1. Participant characteristics according to tobacco exposure during pregnancy

		Total		Group 1		Group 2		Group 3		Group 4		Group 5	
		73,205	100%	31,370	42.9%	12,082	16.5%	15,130	20.7%	11,818	16.1%	2,805	3.8%
		n	%	n	%	n	%	n	%	n	%	n	%
**Antenatal and perinatal backgrounds**													
Maternal age at pregnancy, years													
	<20	463	0.6%	92	0.3%	150	1.2%	51	0.3%	130	1.1%	40	1.4%
	20–29	27,964	38.2%	10,784	34.4%	5,301	43.9%	5,162	34.1%	5,413	45.8%	1,304	46.5%
	30–39	42,179	57.6%	19,285	61.5%	6,221	51.5%	9,344	61.8%	5,958	50.4%	1,371	48.9%
	≥40	2,599	3.6%	1,209	3.9%	410	3.4%	573	3.8%	317	2.7%	90	3.2%
Parity	0	21,718	29.7%	10,354	33.0%	4,253	35.2%	3,635	24.0%	3,012	25.5%	464	16.5%
	1	25,389	34.7%	11,389	36.3%	4,081	33.8%	5,410	35.8%	3,783	32.0%	726	25.9%
	≥2	26,098	35.7%	9,627	30.7%	3,748	31.0%	6,085	40.2%	5,023	42.5%	1,615	57.6%
Sex, male		37,467	51.2%	16,098	51.3%	6,215	51.4%	7,740	51.2%	5,958	50.4%	1,456	51.9%
Gestational age, weeks	≤28	104	0.1%	47	0.1%	13	0.1%	21	0.1%	19	0.2%	4	0.1%
	29–34	761	1.0%	296	0.9%	118	1.0%	174	1.2%	133	1.1%	40	1.4%
	35–36	2,339	3.2%	1,008	3.2%	348	2.9%	468	3.1%	394	3.3%	121	4.3%

	37–41	70,001	95.6%	30,019	95.7%	11,603	96.0%	14,467	95.6%	11,272	95.4%	2,640	94.1%
SGA		2,520	3.4%	1,036	3.3%	404	3.3%	471	3.1%	408	3.5%	201	7.2%
Caesarian section		13,512	18.5%	5,578	17.8%	2,166	17.9%	2,909	19.2%	2,289	19.4%	570	20.3%
Maternal allergy		37,701	51.5%	16,294	51.9%	6,050	50.1%	7,980	52.7%	6,022	51.0%	1,355	48.3%
Alcohol drinking		2,042	2.8%	532	1.7%	266	2.2%	508	3.4%	473	4.0%	263	9.4%
**<u>Socioeconomic background</u>**													
Maternal education, years	<10	2,800	3.8%	255	0.8%	270	2.2%	627	4.1%	1,038	8.8%	610	21.7%
	10–12	23,022	31.4%	6,718	21.4%	4,018	33.3%	5,398	35.7%	5,468	46.3%	1,420	50.6%
	13–16	46,234	63.2%	23,599	75.2%	7,628	63.1%	8,968	59.3%	5,268	44.6%	771	27.5%
	≥17	1,149	1.6%	798	2.5%	166	1.4%	137	0.9%	44	0.4%	4	0.1%
Annual family income, JPY													
	<2,000,000	3,747	5.1%	883	2.8%	698	5.8%	741	4.9%	1,017	8.6%	408	14.5%
	2,000,000–5,999,999	49,522	67.6%	19,956	63.6%	8,168	67.6%	10,758	71.1%	8,614	72.9%	2,026	72.2%
	6,000,000–9,999,999	16,774	22.9%	8,818	28.1%	2,726	22.6%	3,094	20.4%	1,827	15.5%	309	11.0%
	≥10,000,000	3,162	4.3%	1,713	5.5%	490	4.1%	537	3.5%	360	3.0%	62	2.2%

**Postnatal backgrounds**												
Sibling	40,522	55.4%	16,919	53.9%	6,276	51.9%	8,862	58.6%	6,719	56.9%	1,746	62.2%
Breastfeeding at 1 month	72,270	98.7%	31,099	99.1%	11,929	98.7%	14,950	98.8%	11,609	98.2%	2,683	95.7%
Nursery at 1 year	20,003	27.3%	7,397	23.6%	3,754	31.1%	4,026	26.6%	3,812	32.3%	1,014	36.1%
Vaccines												
DPT vaccine	65,981	90.1%	28,647	91.3%	10,803	89.4%	13,652	90.2%	10,461	88.5%	2,418	86.2%
Hib vaccine	70,069	95.7%	30,246	96.4%	11,527	95.4%	14,494	95.8%	11,215	94.9%	2,587	92.2%
Pneumococcal vaccine	68,772	93.9%	29,748	94.8%	11,325	93.7%	14,200	93.9%	10,992	93.0%	2,507	89.4%
Rotavirus vaccine	31,918	43.6%	15,380	49.0%	5,038	41.7%	6,542	43.2%	4,303	36.4%	655	23.4%
Palivizumab	1,907	2.6%	770	2.5%	294	2.4%	415	2.7%	337	2.9%	91	3.2%
**Infection outcomes**												
Infections	34,839	47.6%	14,584	46.5%	5,831	48.3%	7,235	47.8%	5,780	48.9%	1,409	50.2%
CNSI	110	0.2%	37	0.1%	20	0.2%	28	0.2%	20	0.2%	5	0.2%
OM	8,638	11.8%	3,422	10.9%	1,506	12.5%	1,783	11.8%	1,520	12.9%	407	14.5%
URTI	23,439	32.0%	10,039	32.0%	3,950	32.7%	4,829	31.9%	3,739	31.6%	882	31.4%
LRTI	10,774	14.7%	4,273	13.6%	1,791	14.8%	2,228	14.7%	1,956	16.6%	526	18.8%
GI	6,899	9.4%	2,572	8.2%	1,189	9.8%	1,478	9.8%	1,320	11.2%	340	12.1%

UTI	548	0.7%	243	0.8%	95	0.8%	104	0.7%	90	0.8%	16	0.6%

CNSI, central nervous system infection; DPT: diphtheria, pertussis, and tetanus; GI: gastrointestinal infection; Hib: *Haemophilus influenzae* type b; JPY, Japanese yen; LRTI: lower respiratory tract infection; OM: otitis media; SGA: small for gestational age; URTI: upper respiratory tract infection; UTI: urinary tract infection.

Pregnant women were categorized into five groups based on their tobacco exposure: "Never smoked without exposure to SHS (Group 1)," "Never smoked with exposure to SHS (Group 2)," "Quit smoking without exposure to SHS (Group 3)," "Quit smoking with exposure to SHS (Group 4)," and "Current smoker with/without exposure to SHS (Group 5)."

Infections include CNSI, OM, URTI, LRTI, GI, and UTI.
